# Pharmacokinetic Study of Delavinone in Mice after Intravenous and Oral Administration by UPLC-MS/MS

**DOI:** 10.1155/2019/3163218

**Published:** 2019-03-21

**Authors:** Shuanghu Wang, Zhiguang Zhang, Zheng Yu, Cheng Han, Xianqin Wang

**Affiliations:** ^1^The Laboratory of Clinical Pharmacy, The People's Hospital of Lishui, Lishui 323000, China; ^2^Analytical and Testing Centre, School of Pharmaceutical Sciences, Wenzhou Medical University, Wenzhou 325035, China

## Abstract

Thirty-one compounds, including delavinone, were isolated from the methanol extract of* F. cirrhosa* by modern chromatographic techniques. The pharmacological action of* Fritillaria* is widely used in clinical practice. However, the pharmacokinetic studies on delavinone have not been reported. Therefore, the chemical constituents of this species were investigated. Therefore, it is necessary to establish an analytical method to monitor the concentration of delavinone. An UPLC-MS/MS method was established to determine delavinone in the mouse blood, and the pharmacokinetics of delavinone after intravenous (1.0 mg/kg) and intragastric (2.5, 10.0 mg/kg) administration were studied. The lower limit of quantification was 1.0 ng/mL. The intraday and interday precision RSD were less than 13%, the accuracy ranged from 96.8% to 104.9%, the average recovery was better than 80.6%, and the matrix effect was between 88.8% and 103.4%. The UPLC-MS/MS method has been successfully applied to the pharmacokinetics of delavinone in mice. The noncompartment model was used to fit the main pharmacokinetic parameters. It was found that AUC in mice was higher than that in mice given orally, and the bioavailability of delavinone was 12.4%.

## 1. Introduction

*Fritillaria* has been used in China with a long history of two thousand years, which was documented in the oldest materia medica book “Shen Nong's Classic of Materia Medica” [1, 2]. The genus* Fritillaria* includes 130 species worldwide and is mainly distributed in temperate regions of the northern hemisphere [3, 4]. Most species are distributed in Cyprus, Iran, and Turkey. In traditional Chinese medicine, the bulbs of many* Fritillaria* species (“Beimu” in Chinese) have been used as antitussive, antiasthmatic, and expectorant agents for more than two thousand years [5, 6]. In order to find new natural products or active ingredients, clarify the material basis of* Fritillaria cirrhosa*, provide the basis for the full utilization and rational development of* Fritillaria* medicinal plant resources, and establish a scientific quality evaluation method, the chemical constituents of* Fritillaria cirrhosa* D. Don and Fritillaril delavayi Franch, two origin plants of “Chuanbeimu” in China, have been systematically studied in some literatures, and the determination methods of alkaloids and nucleosides were established [7, 8]. The quality evaluation method of* Fritillaria* was preliminarily discussed, which provided scientific basis for the quality control and variety identification of medicinal species of* Fritillaria*. Thirty-one compounds, including delavinone, were isolated from the methanol extract of* F. cirrhosa* by modern chromatographic techniques. The pharmacological action of* Fritillaria* is widely used in clinical practice. However, the pharmacokinetic studies on delavinone have not been reported. Therefore, the chemical constituents of this species were investigated. Therefore, it is necessary to establish an analytical method to monitor the concentration of delavinone.

To our knowledge, the pharmacokinetics of delavinone have not been reported. In this paper, UPLC-MS/MS method was established to determine delavinone in mouse blood, and the pharmacokinetics of delavinone after intravenous and intragastric administration were studied, and the absolute bioavailability was obtained.

## 2. Materials and Methods

### 2.1. Chemicals and Animal

**Figure 1:**
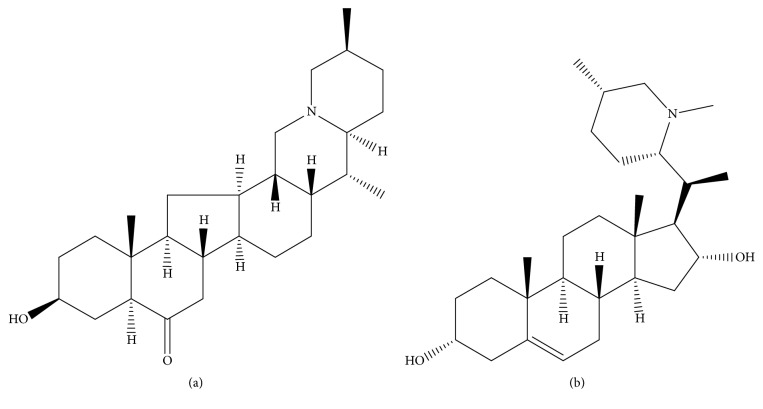
Chemical structure of delavinone (a) and hapepunine (IS, (b)).

Delavinone (purity >98%, Figure 1(a)) and hapepunine (internal standard (IS), purity >98%, Figure 1(b)) were purchased from Chengdu Mansite Pharmaceutical Co., Ltd. Ultrapure water was prepared by the Millipore Milli-Q purification system (Bedford, MA, USA). HPLC-grade acetonitrile and methanol were purchased from Merck Co., Ltd. (Darmstadt, Germany). ICR mice (weight 20-22 g) were purchased from the Animal Experimental Center of Wenzhou Medical University.

### 2.2. Instrument and Condition

ACQUITY I-Class UPLC and XEVO TQS-micro Triple Quadrupole Mass Spectrometer (Waters Corp, Milford, MA, USA) were used. Masslynx 4.1 software (Waters Corp.) was used for data acquisition and instrument control.

The mobile phase consisted of acetonitrile and 0.1% formic acid with a gradient elution at a flow rate of 0.4 mL/min, as well as 0-0.2 min, 0.1% formic acid 90%; 0.2-1.5 min, 0.1% formic acid 90%-15%; 1.5-2.0 min, 0.1% formic acid 15%; 2.0-2.5 min, 0.1% formic acid 15%-90%; and 2.5-4.0 min, 0.1% formic acid 90%. The BEH C18 (2.1 mm × 50 mm, 1.7 *μ*m) was used as UPLC column set to 30°C.

**Figure 2:**
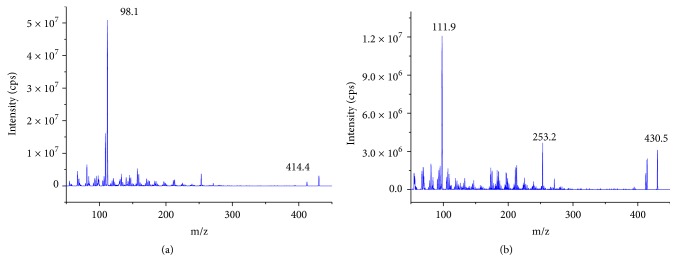
Mass spectrum of delavinone (a) and hapepunine (IS, (b)).

The capillary voltage was set to 2.0 kV, the ion source temperature was 150°C, and the desolvation temperature was 400°C. Nitrogen was used as the desolvation gas (800 L/h) and the cone gas (50 L/h). The MRM model was quantitatively analyzed for delavinone m/z 414.4→98.1 and IS m/z 430.5→111.9 in ESI positive interface (Figure 2).

### 2.3. Standard Curve Preparation

A stock solution of delavinone (1.0 mg/mL) and IS (1.0 mg/mL) was prepared with methanol-water (50:50). Working solutions were prepared by diluting with a delavinone stock solution in methanol. A working solution of the IS 50 ng/mL was prepared by diluting with a IS stock solution with acetonitrile.

The blank mouse blood was spiked with an appropriate amount of standard working solution to prepare the blood standard curve of delavinone (1, 5, 20, 50, 200, and 500 ng/mL). Quality Control (QC) samples were prepared in the same manner as the standard curve (1, 3, 180, and 450 ng/mL).

### 2.4. Sample Preparation

20 *μ*L of blood sample was added to 1.5 mL Eppendorf tube; then 100 *μ*L of acetonitrile (containing IS of 50 ng/mL) was added, vortexed for 1.0 min, and centrifuged at 4°C for 1 min at 13000 rpm. 80 *μ*L of the supernatant was taken and 2 *μ*L injected into UPLC-MS/MS for analysis [9].

### 2.5. Pharmacokinetics

Eighteen mice were randomly divided into three groups: one group was intravenous administration (1.5 mg/kg), and two groups were intragastric administration (2.5, 10 mg/kg), with 6 rats in each group. Then 20 *μ*L of blood was obtained in a 1.5 mL Eppendorf tube from tail vein at 0.0833, 0.5, 1, 1.5, 2, 3, 4, and 8 h after administration in mice and frozen at -20°C.

DAS 2.0 software (China Pharmaceutical University) used a noncompartmental model to fit pharmacokinetic parameters.

## 3. Results

### 3.1. Method Validation

**Figure 3:**
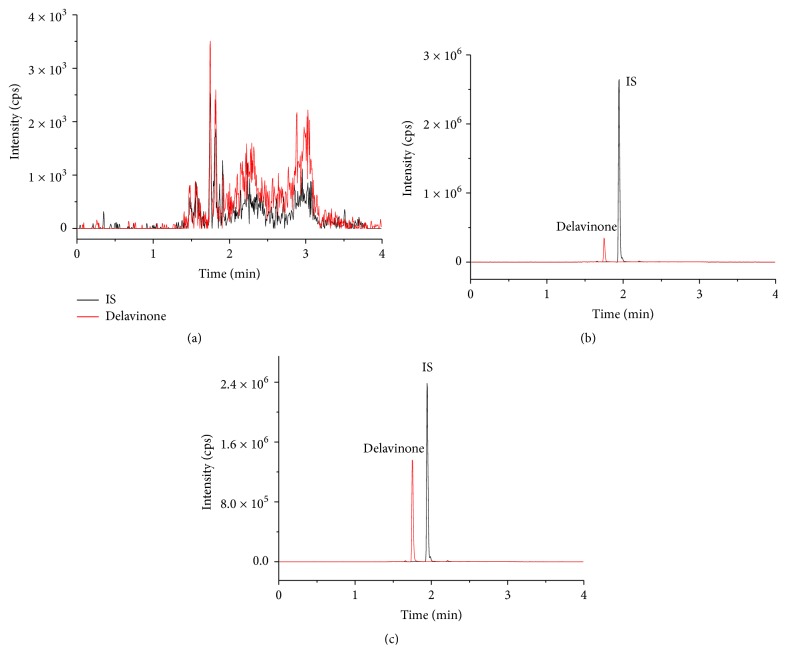
UPLC-MS/MS of delavinone and hapepunine (IS) in mouse blood; (a) blank blood; (b) blank blood spiked delavinone and IS; (c) a mouse blood sample.

Figure 3 shows that the retention times of delavinone and IS were 1.75 and 1.94 min, respectively. No obvious endogenous substances interfered with the detection.

The standard curve equation of delavinone in the mouse blood was the following: Y=0.0052C+0.0040, r= 0.9982, where Y represents the ratio of the peak area of delavinone and IS and C represents the concentration of delavinone in mouse blood. The LLOQ in the mouse blood is 1.0 ng/mL; the signal-to-noise ratio is 8.

The intraday precision RSD was less than 12%, the interday precision RSD was less than 13%, the accuracy ranged from 96.8% to 104.9%, the matrix effect was between 88.8% and 103.4%, and the average recovery was better than 80.6% in Table 1.

In the mouse blood at room temperature 2h, -20°C for 30 days, and freeze-thaw stability test, the variation of delavinone was within ± 12%; RSD was less than 11%, indicating that the stability of delavinone was acceptable.

### 3.2. Pharmacokinetics Study

**Figure 4:**
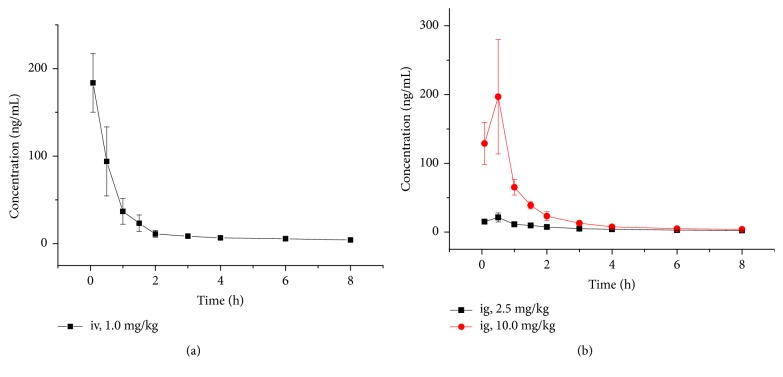
Time-blood concentration curve of delavinone in mouse blood after intravenous (1.0 mg/kg) and oral (2.5, 10 mg/kg) administration.

The concentration-time curve of delavinone is shown in Figure 4. The main pharmacokinetic parameters are shown in Table 2. As can be seen from Table 2, the average absolute bioavailability of delavinone was 12.4%.

## 4. Discussion

Compared with LC-MS/MS, UPLC-MS/MS is more sensitive and has obvious advantages in the study of pharmacokinetics [10–12]. At the same time, its powerful separation and analysis ability is suitable for the metabolism of complex components of traditional Chinese medicine and complex compound systems in vivo. A UPLC-MS/MS method was established for the determination of delavinone in mice blood. The pharmacokinetics of delavinone in mice after intravenous and intragastric administration were studied, and the bioavailability of delavinone was obtained.

The choice of positive and negative electrodes for electrospray ESI is often evaluated in methodology [13, 14]. As an alkaloid and alkaline compound, delavinone is more suitable for ESI positive electrode detection. Our experiments also show that ESI positive ion mode is more sensitive than negative ion mode. We optimized ionization conditions of delavinone and IS, and the highest abundance of delavinone fragment was m/z 98.1; the highest abundance of IS fragment ion was m/z 111.9. Therefore, the quantitative analysis was m/z 414.4→98.1 for delavinone (cone voltage 30v, collision energy 24v) and m/z 430.5→111.9 for IS (cone voltage 30v, collision energy 23v). Methods selection of IS was also an important task in the process of establishment. Finally, hapepunine was selected as IS because it and delavinone have similar structure, retention time, and mass spectrometric ionization mode.

As far as possible, the internal interfering substances are separated from the retention time by HPLC; mobile phase and chromatographic column determine the chromatographic behavior [15, 16]. We tried different chromatographic columns such as BEH C18 (2.1 mm×50 mm, 1.7 *μ*m), BEH C18 (2.1 mm×100 mm, 1.7 *μ*m), and HSS T3 (2.1 mm×100mm,1.8*μ*m). The results showed that BEH C18 (2.1 mm×50 mm, 1.7 *μ*m) had the best peak time and peak effect. We tried acetonitrile-0.1% formic acid, acetonitrile-10 mmol/L ammonium acetate solution (containing 0.1% formic acid), methanol-0.1% formic acid, methanol-10 mmol/L ammonium acetate solution (containing 0.1% formic acid), and gradient elution. The results showed that acetonitrile-0.1% formic acid obtained the most satisfactory chromatographic peak shape and retention time. So BEH C18 (2.1 mm *∗*50 mm, 1.7 micron) column and acetonitrile-0.1% formic acid as mobile phase were used in this work.

Before UPLC-MS/MS analysis, removing protein and potential interference is a key point of method establishment [17–20]. We tried ethyl acetate, chloroform, and ethyl ether for liquid extraction and also tried methanol, acetonitrile, and methanol-acetonitrile (1:1, v/v) for direct precipitation method and finally found that acetonitrile precipitation method was the best, followed by ethyl acetate liquid extraction method. Direct acetonitrile precipitation method is fast and simple and it also shows good recovery and acceptability of matrix effect, so this study used acetonitrile precipitation method as a blood sample treatment.

UPLC-MS/MS has been applied to the quantitative determination of delavinone in blood, which is faster and more sensitive than traditional HPLC. Only 4 min can complete the analysis of blood samples, which can save a lot of time and solvent. In addition, the LLOQ (1ng/mL) of delavinone is relatively low, which could be used to determine a lower blood concentration at the final sampling point. A UPLC-MS/MS method was established to study the pharmacokinetics of delavinone in mice after sublingual injection or intragastric administration. Mice have less blood, so only 20*μ*L of blood was taken at each point of time. The blood of mice was directly treated by acetonitrile precipitation method, and the concentration of delavinone in whole blood was determined by UPLC-MS/MS. There, the concentration after 8h was too low to be determined (lower than LLOQ of 1 ng/mL); therefore the data of 12 h and 24 h was not shown. No literature has been reported on the pharmacokinetics of delavinone in rats or mice. The noncompartment model was used to fit the main pharmacokinetic parameters. It was found that AUC in mice was higher than that in mice given orally, and the bioavailability of delavinone was 12.4%.

## 5. Conclusion

This study developed a sensitive and rapid UPLC-MS/MS method for the determination of delavinone in mouse blood with the range of 1.0-500 ng/mL. This method was successfully applied to the pharmacokinetics of delavinone in mice, and the absolute bioavailability was 12.4%.

## Figures and Tables

**Table 1:** Accuracy, precision, matrix effect, and recovery of delavinone in the mouse blood.

Concentration	Accuracy (%)		Precision (RSD%)		Metrix effect	Recovery
(ng/mL)	Intraday	Interday	Intraday	Interday	(%)	(%)
1	104.9	96.8	11.9	12.4	90.5±4.9	92.5±3.4
3	102.1	98.7	9.4	9.6	88.8 ±7.9	84.1±7.5
180	97.4	102.9	5.0	2.7	99.0±4.6	80.6±3.7
450	96.8	101.3	6.3	7.6	103.4 ±7.5	84.2±8.1

**Table 2:** Main pharmacokinetic parameters of delavinone in mice.

Parameters	Unit	iv (1.0 mg/kg)	ig (2.5 mg/kg)	ig (10 mg/kg)
AUC_(0-t)_	ng/mL*∗*h	169.8±44.2	48.0 ±7.0	229.7 ±49.6
AUC_(0-*∞*)_	ng/mL *∗*h	245.2 ±106.1	60.8 ±12.4	241.4 ±52.5
MRT_(0-t)_	h	1.4 ±0.1	2.5 ±0.2	1.4 ±0.2
MRT_(0-*∞*)_	h	9.2 ±6.3	4.8 ±1.0	1.9 ±0.4
t_1/2z_	h	9.5 ±5.2	4.1 ±0.9	2.7±0.8
CL_z/F_	L/h/kg	4.7 ±1.8	42.6 ±8.8	43.0±8.6
V_z/F_	L/kg	46.4 ±31.9	242.2 ±36.0	162.3±51.3
C_max_	ng/mL	183.7 ±33.5	21.3 ±6.5	196.9±83.3
Bioavailability			11.3%	13.5%

## Data Availability

The data used to support the findings of this study are included within the article.
